# When the Midline Diastema Is Not Characteristic of the “Ugly Duckling” Stage

**DOI:** 10.1155/2015/924743

**Published:** 2015-08-05

**Authors:** Orlando Motohiro Tanaka, Alessandro Yuske Kusano Morino, Oscar Fernando Machuca, Neblyssa Ágatha Schneider

**Affiliations:** ^1^Graduate Dentistry Program, Orthodontics, School of Health and Biosciences, Pontifícia Universidade Católica do Paraná, Rua Imaculada Conceição 1155, 80215-901 Curitiba, PR, Brazil; ^2^Undergraduation Dentistry Course, Pontificia Universidade Católica do Paraná, 80215-901 Curitiba, PR, Brazil; ^3^Graduate Dentistry Program, Orthodontics, Pontificia Universidade Católica do Paraná, 80215-901 Curitiba, PR, Brazil

## Abstract

This case report presents the interceptive orthodontic treatment of a 9-year, 5-month-old boy with class I malocclusion, a 9.0-mm maxillary midline diastema, and deviation from the midline. The treatment goals were to decrease the magnitude of the diastema and to simulate the characteristics of the “ugly duckling” stage. Braces were placed on the first molars and the maxillary central incisors. The biomechanics of the anchors on the first molars elicited substantial mesial movement of the left central incisor to match the midline. A flat wire segment was bonded onto the palatal surface of the central incisors for retention.

## 1. Introduction

A midline diastema is typically part of normal dental development during the period of mixed dentition [1]. However, several factors can cause a diastema that may require an intervention.

Enlarged labial frena have been blamed for the majority of persistent diastemas, but the etiologic role of this structure is now understood to influence only a small proportion of cases. Other etiologies associated with diastema include oral habits, muscular imbalances, physical impediments, abnormal maxillary arch structure, and various dental anomalies [2].

Supernumerary teeth, which occur in both the primary and permanent dentitions, cause a variety of pathological disturbances [3], the most common of which is midline diastema. In the periods of mixed and early permanent dentitions, median diastemas can be transient or created by developmental, pathological, or iatrogenic factors and are a major aesthetic concern for patients and/or their parents [4].

The presence of a diastema between the teeth is a common feature of the anterior dentition that remains until the completion of the permanent dentition [1]. Carefully developed diagnoses and advanced planning enable the identification of the most appropriate treatment to address the needs of each individual patient [5].

There has been much debate regarding the ideal time to initiate orthodontic treatment, and from the orthodontist's perspective, only maxillary midline diastemas and congenitally missing teeth should be treated in later stages [6].

An effective diastema treatment requires the correct diagnosis of its etiology and an intervention that is relevant to that specific etiology, including medical and dental histories, radiographic and clinical examinations, and possibly tooth-size evaluations [2].

The needs for treatment are primarily attributed to aesthetic and psychological rather than functional reasons. Although it is often the case, treatment plans should not be selected empirically but should rather be based on adequate scientific documentation. The ideal treatment should deal not only with the diastema but also with the cause of the diastema. Irrespective of the selected treatment, the permanent retention of stable results should be considered to be a treatment objective [7].

Many different techniques can be used for diastema closure. Some of the methods that have been suggested for the closure of unaesthetic diastemas involve the use of fixed or removable appliances, elastics, composite build-ups, and brass wires that are placed in the interdental spaces distal to the central incisors to form a loop that is tightened by twisting the ends together gently until the diastema is closed [8].

The aim of this case report was to underscore the orthodontic biomechanics that were applied to minimize a very large maxillary midline diastema and to illustrate the clinical results.

## 2. Case Presentation

**Figure 1:**
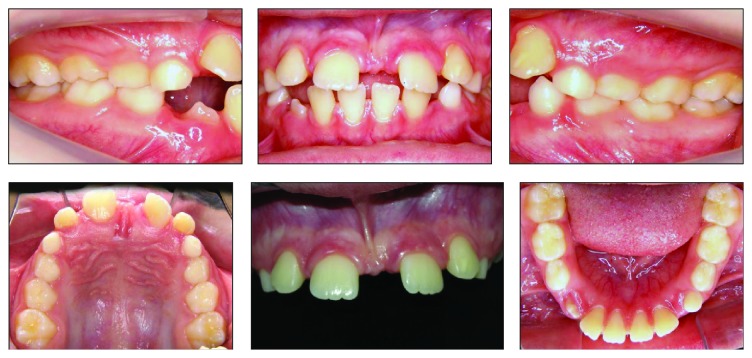
Pretreatment intraoral photographs.

**Figure 2:**
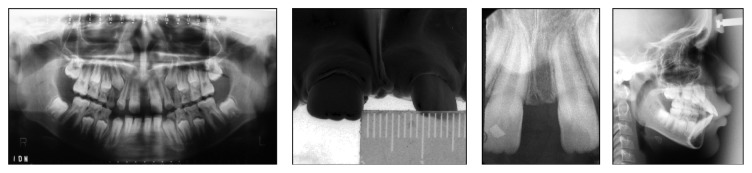
Pretreatment radiographs.

**Figure 3:**
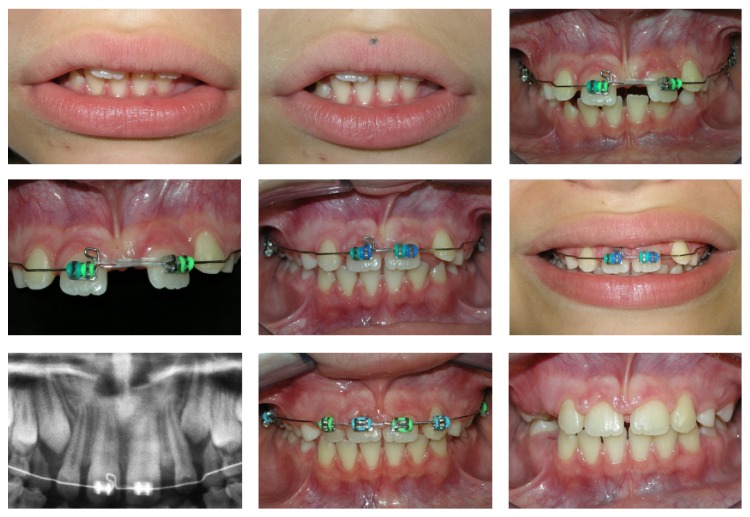
Progress. Determination of the correct midline and biomechanics for moving the maxillary right central incisor to the left.

A 9-year, 5-month-old male patient came for a consultation with the chief complaint that he did not like the gap between his maxillary central incisors because it resulted in his exposure to bullying at school. On examination, the following factors were revealed: class I malocclusion, overjet of 4.0 mm, mild overbite, diastema between the central incisors of 9 mm (the mother reported that the extraction of a mesiodens was performed at the age of 8), diastemas between the central and lateral incisors and between the lower incisors, a labial frenulum with a low insertion, and rotation of the incisors (Figures 1 and 2). The correct mandibular midline was diagnosed with consideration of the maxillary midline via the “V” cupid bow technique [9] (Figure 3).

Radiographically, the correct sequence of eruption, skeletal class I malocclusion, a sagittal growth trend, and proclination of the maxillary and mandibular incisors were noted (Figure 2).

The diagnosis and treatment options for minimizing the width of the diastema were explained to the patient, his parents, and the general practitioner who referred the patient.

### 2.1. Treatment Objectives

The goal of the orthodontic treatment was to reduce the size of the diastemas, particularly those between the maxillary central and left lateral incisors. At this stage, we sought to maintain the positions of the upper and lower incisors.

### 2.2. Treatment Alternatives

The following alternatives were explained: (a) a removable appliance with clasps and digital springs positioned distal to the left central incisors and associated with a labial arch, which was not indicated because it would only tip the crown and would not result in body movement; (b) brackets bonded to the maxillary central incisors to reciprocally move the incisors with elastic chains, which would deviate the midline to the left; and (c) partial fixed appliances with brackets bonded to the incisors and bands on the first permanent molars to move the left central incisor using the teeth on the right side or the archwire as an anchorage point. This latter approach was applied.

### 2.3. Treatment Progress

Orthodontic treatment was initiated with the cementation of the first molar bands and bonding brackets on the maxillary central incisors. The treatment began with a .016 × .022-in stainless steel arch with a fair loop flush on the mesial side of the right central incisor bracket. Omega loops were kept tight to the tubes of the molars. An elastic chain connected the flap mesial to the left central incisor to the loop to move the left incisor toward the right central incisor. After achieving a position similar to the “ugly duckling” stage, the bracket was bonded to the left lateral incisor and moved mesially with elastics.

### 2.4. Treatment Results

**Figure 4:**
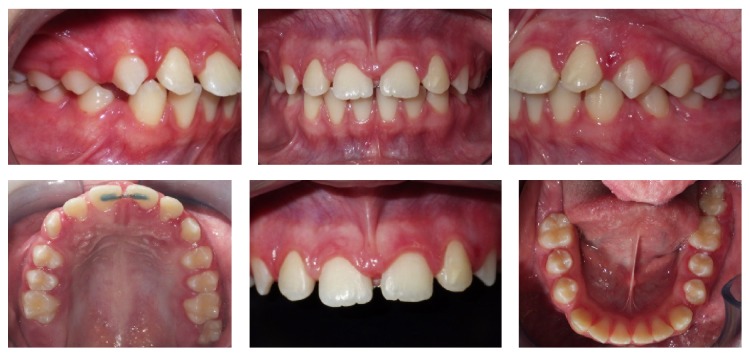
Posttreatment intraoral photographs.

**Figure 5:**
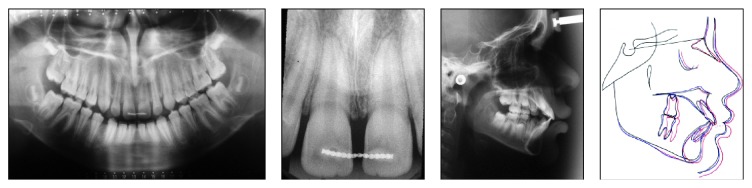
Posttreatment radiographs and cephalometric measurements.

The treatment promoted a reduction in the diastema to a size that was similar to the physiological “ugly duckling” phase. The maxillary left central and lateral incisors were moved mesially to a greater extent than the maxillary right central incisor to match the midline. The appliance was removed, and a flat wire segment was bonded into the palatal surfaces of both central incisors with the intention of being maintained until complete eruption of the canines (Figures 4 and 5).

## 3. Discussion

In dentistry, the challenge is not only to produce good occlusion but also to obtain good occlusion with good aesthetic results. In the mixed dentition, the maxillary midline diastema is physiological. However, the size of the diastema should be considered.

The midline diastema has a multifactorial etiology. In addition to the labial frenulum, microdontia, mesiodens, peg-shaped lateral incisors, lateral incisor agenesis, cysts in the midline region, habits such as finger sucking, tongue thrusting and/or lip sucking, dental malformations, genetics, maxillary incisor proclination, dental-skeletal discrepancies, and imperfect coalescence of the interdental septum should be considered as factors that can cause diastema.

The clinical diagnosis is important and should necessarily include radiographic examinations. During the “ugly duckling” stage, the long axes of the roots of the maxillary central and lateral incisors diverge from each other [10], which often misleads practitioners to a diagnosis of a diastema caused by a hypertrophic labial frenulum [11].

Before the practitioner can determine the optimal treatment, he or she must consider the contributing factors. These factors include normal growth and development, tooth-size discrepancies, excessive incisor vertical overlap due to different causes, mesiodistal and labiolingual incisor angulations, generalized spacing, and pathological conditions [12].

Small (2 mm or less) but nonaesthetic diastemas can be closed by tipping the central incisors mesially with removable appliances. In the majority of such cases, the use of an elastic that involves both central incisors causes no problems. However, if the rubber band slides into the soft tissues, it is difficult if not impossible to retrieve it, and the elastic will continue along the distal surfaces of the roots to destroy the periodontal attachments and produce inflammation [13]. Behrents noted that the use of elastics for orthodontic tooth movement can be advantageous, but their use is also associated with disadvantages and risks that should be known and considered by practitioners and consumers alike [14].

However, large diastemas, such as that described in the present case report, require braces and the application of biomechanical force. Extra attention should be provided when attempting to move the lateral incisors mesially due to the proximity to the crowns of the canines.

Large maxillary midline diastemas such as that described in the present case report are not common situations in patients at the age of nine years. This patient was bullied at school, and minimizing the size of the diastema provided self-esteem and confidence to the patient. The anchorage applied in the biomechanics consisted of the use of archwires that were flush to the molar tubes and an elastic chain that engaged the boot-loop bend mesial to the bracket of the right central incisor. To simulate the “ugly duckling” stage, the midline was not completely closed. The patient and his parents were informed of the necessity of a phase II orthodontic treatment.

The differential diagnosis led to the treatment approach that most effectively addressed the patient's problem. By treating the causes of diastemas rather than only the spaces, dentists can enhance both the dental function and appearance of the patient.

Fixed orthodontic retention is the most appropriate type of retention when a maxillary midline diastema is mechanically closed. In cases of deep overbite, bonding with a different retention wire design may be recommended [15]. Due to the possibility of relapse, retention must always be considered in either interceptive or corrective treatment, regardless of how carefully the space was initially handled.

## 4. Conclusion

The biomechanical forces applied via fixed appliances anchored on the first permanent molars provided midline diastema closure to an extent that was characteristic of the “ugly duckling” stage. The aesthetic goals were achieved, and the patient's complaints were resolved.
